# Mitochondria-targeted antioxidant SKQ1 protects cornea from oxidative damage induced by ultraviolet irradiation and mechanical injury

**DOI:** 10.1186/s12886-018-0996-7

**Published:** 2018-12-27

**Authors:** Evgeni Yu. Zernii, Olga S. Gancharova, Veronika V. Tiulina, Andrey A. Zamyatnin, Pavel P. Philippov, Viktoriia E. Baksheeva, Ivan I. Senin

**Affiliations:** 1 Belozersky Institute of Physico-Chemical Biology, Lomonosov Moscow State University, Moscow, 119992 Russia; 2 Institute for Regenerative Medicine, Sechenov First Moscow State Medical University, Moscow, 119991 Russia; 3 Institute of Molecular Medicine, Sechenov First Moscow State Medical University, Moscow, 119991 Russia

**Keywords:** Photorefractive surgery, Iatrogenic ocular damage, UV-induced oxidative stress, Cornea, SkQ1

## Abstract

**Background:**

Cornea protects the eye against natural and anthropogenic ultraviolet (UV) damage and mechanical injury. Corneal incisions produced by UV lasers in ophthalmic surgeries are often complicated by oxidative stress and inflammation, which delay wound healing and result in vision deterioration. This study trialed a novel approach to prevention and treatment of iatrogenic corneal injuries using SkQ1, a mitochondria-targeted antioxidant approved for therapy of polyethiological dry eye disease.

**Methods:**

Rabbit models of UV-induced and mechanical corneal damage were employed. The animals were premedicated or treated with conjunctival instillations of 7.5 μM SkQ1. Corneal damage was assessed by fluorescein staining and histological analysis. Oxidative stress in cornea was monitored by measuring malondialdehyde (MDA) using thiobarbituric acid assay. Total antioxidant activity (AOA) was determined using hemoglobin/H_2_O_2_/luminol assay. Glutathione peroxidase (GPx) and superoxide dismutase (SOD) activities were measured using colorimetric assays.

**Results:**

In both models corneas exhibited fluorescein-stained lesions, histologically manifesting as basal membrane denudation, apoptosis of keratocytes, and stromal edema, which were accompanied by oxidative stress as indicated by increase in lipid peroxidation and decline in AOA. The UV-induced lesions were more severe and long healing as corneal endothelium was involved and GPx and SOD were downregulated. The treatment inhibited loss of keratocytes and other cells, facilitated re-epithelialization and stromal remodeling, and reduced inflammatory infiltrations and edema thereby accelerating corneal healing approximately 2-fold. Meanwhile the premedication almost completely prevented development of UV-induced lesions. Both therapies reduced oxidative stress, but only premedication inhibited downregulation of the innate antioxidant activity of the cornea.

**Conclusions:**

SkQ1 efficiently prevents UV-induced corneal damage and enhances corneal wound healing after UV and mechanical impacts common to ocular surgery. Its therapeutic action can be attributed to suppression of mitochondrial oxidative stress, which in the first case embraces all corneal cells including epitheliocytes, while in the second case affects residual endothelial cells and stromal keratocytes actively working in wound healing. We suggest SkQ1 premedication to be used in ocular surgery for preventing iatrogenic complications in the cornea.

## Background

Cornea is the quickly regenerating defensive barrier of the eye, protecting intraocular structures from environmental stress. In particular, cornea absorbs ultraviolet (UV) component of sunlight and prevents it from reaching vulnerable parts of the eye, such as the retina [1]. Meanwhile, prolonged UV irradiation of the eye surface is associated with high risk of corneal injury. It is widely regarded that UV-induced damage is mediated by oxidative stress in corneal cells (for review, see [2]). To endure this exposure, cornea possesses innate antioxidant qualities [3]. While sunlight is the major source of UV rays, in some cases human eyes are exposed to more harmful forms of UV, as, for instance, during medical procedures. Indeed, UV lasers of different wavelengths have wide range of applications in ophthalmology. Far-ultraviolet excimer lasers (150–200 nm) allow evaporating corneal epithelium and stroma and making cuts of precise depth and shape without thermal damage to the adjacent tissue [4]. Thereby, they are utilized in vision correction surgeries, such as laser-assisted in situ keratomileusis (LASIK) and photorefractive keratectomy (PRK) [5]. In both operations excimer laser is employed to remodel the corneal stroma. However, in LASIK, an epithelium flap is temporally created at the site of the operation using either microtome or femtosecond infrared laser and it is put in place after the procedure. In PRK, the epithelium is completely removed mechanically, chemically or with application of excimer laser (transepithelial PRK) [6]. Longer wavelength UV lasers (200–350 nm) are less commonly used to operate on cornea, as they are known to produce irregular cut edges and have varied mutagenic effects on epithelial cells [7–10].

Even relatively mild irradiations with UV light of different wavelengths can be detrimental to corneal structure and function as they result in elevation of intracellular reactive oxygen species (ROS) [11–14]. Excessive ROS accumulation leads to oxidative damage to proteins and membranes as well as nuclear and mitochondrial DNA [11, 12]. Mitochondria are mostly sensitive to oxidative stress as they are the major source of the basic ROS levels and depend on redox processes in respect to their functions, biogenesis and turnover [11]. Under intense UV irradiation, the antioxidant mechanisms of the cornea can be overwhelmed, resulting in oxidative damage to mitochondria and other cellular components thereby promoting apoptosis of corneal cells and inducing ocular pathology [11, 12]. Oxidative stress triggers inflammation and deters corneal wound healing, which increases risk of visual complications, as quick restoration of corneal structure after injury is crucial for preservation of its transparency and curvature. Persistent corneal epithelium defects are strongly associated with excessive apoptosis of stromal keratocytes, corneal fibroblasts responsible for remodeling of extracellular matrix and production of growth factors that stimulate proliferation of corneal epithelial cells [15, 16]. Oxidative stress was demonstrated to target both keratocytes and multipotent cells of corneal epithelium, ultimately leading to scarring and opacification of cornea and irreversible deterioration of vision [17].

In view of this, there is a growing interest in applying antioxidants to prevent and treat iatrogenic complications in cornea associated with UV irradiation. A group of antioxidants capable of bypassing plasma membrane and accumulating in mitochondria emerged in recent decades and they were shown to outperform conventional antioxidants in terms of specificity and activity levels [18–21]. Among these mitochondria-targeted antioxidants, plastoquinonyl-decyl-triphenylphosphonium bromide (SkQ1) was approved for treating multi-etiological dry eye syndrome (DES) [22, 23]. Recently, using rabbit (*Oryctolagus cuniculus*) model of anesthesia-induced DES we have demonstrated that SkQ1 at dosage 7.5 μM not only suppresses oxidative stress, but also enhances intrinsic antioxidant defense of the corneal cells thereby exhibiting pronounced cornea-protecting activity [24].

In this study, using rabbit models involving UV irradiation and corneal scarification, we trialed feasibility of SkQ1 for prevention and treatment of UV-induced and mechanical corneal injury associated with common ophthalmological procedures. The benefits of mitochondria-targeted antioxidant therapy using SkQ1 were assessed by means of clinical and histological analysis as well as biochemical assays. The mechanisms underlying effects of SkQ1 on corneal state and functionality as well as prospective ophthalmological applications of the mitochondria-targeted antioxidant therapy are considered.

## Methods

### Materials

SkQ1 (10-(6′-plastoquinonyl)-decyltriphenylphosphonium) was provided by the Institute of Mitoengineering of Moscow State University (Moscow, Russia). Anesthetic preparation containing 50 mg/ml tiletamine and 50 mg/ml zolazepam was bought from Virbac, UK. Fluorescein sodium solution was purchased from Novartis, Switzerland. Superoxide dismutase and malondialdehyde assay kits were from Sigma-Aldrich, USA. Glutathione peroxidase assay kit was from Randox, UK. Reagents and supplies for histology were from Biovitrum, Russia. Other reagents were from Gibco, Sigma-Aldrich, Amresco and Serva and were at least reagent grade. All buffers and other solutions were prepared using ultrapure deionized water.

### Experimental animals and ethics statement

The study involved a total of 144 pigmented male rabbits (6 months old, 2.3 to 3 kg). The animals were purchased from a certified farm (Krolinfo, Russia). The rabbits were housed individually in 795x745x1776 mm cages at a 12 h light-dark cycle (8:00–20:00) at a temperature of 22–25 °C and humidity of 55–60% with free access to maintenance rabbit food (BioPro, Russia) and water. The health status of all animals was monitored daily and no adverse events were observed during the course of the study. The treatment of the animals was performed according to the 8th edition “Guide for the Care and Use of Laboratory Animals” of the National Research Council and “Statement for the Use of Animals in Ophthalmic and Visual Research” of The Association for Research in Vision and Ophthalmology (ARVO). The protocol was approved by the Belozersky Institute of Physico-chemical Biology Animal Care and Use Committee (Protocol number 1/2016). For biochemical and histological studies of the cornea, the rabbits were humanely euthanized by introduction into general anesthesia and subsequent intracardiac injection of the 1 ml of 20 mg/ml xylazine hydrochloride. Enucleating of the eyeballs and corneal excision were performed post-mortem.

### Experimental models and treatment regimen

The experiments were performed using a single-blind method. The animals were allowed to acclimatize to the research facility for 5–7 days before the start of the experiment. All experimental procedures were carried out in the laboratory. During the UV irradiation and trephination sessions, the animals were placed in a restraining device and anesthetized with intramuscular injection of 1:2 mixture of 50 mg/ml tiletamine/zolazepam and 20 mg/ml xylazine hydrochloride (max. 2.2 mg/kg tiletamine/zolazepam, 1.7 mg/kg xylazine hydrochloride). The UV illuminations of the cornea were performed with halogen lamp (312 nm, 8 W) from the distance of 40 cm for 4 days, 20 min per day. Scarification of the cornea was performed using 7 mm circular trephine knife with incisions depth of 50 μm. After the procedure the animals were returned to the housing. For antioxidant treatment (administration Scheme 1), the animals were medicated by conjunctival instillations of 50 μl of either 7.5 μM SkQ1 in vehicle solution (7 mM phosphate buffer saline (PBS) pH 7.4, 0.0001% benzalkonium chloride) or placebo (vehicle solution) 3 times a day for 1–30 days (1–4 days in case of the scarification model), starting immediately after the final irradiation session/trephination. Antioxidant premedication (administration Scheme 2) was performed 60 min before the irradiation session by conjunctival instillations of 7.5 μM SkQ1 or placebo, 1 instillation each 10 min.

### Division of groups

**Fig. 1:**
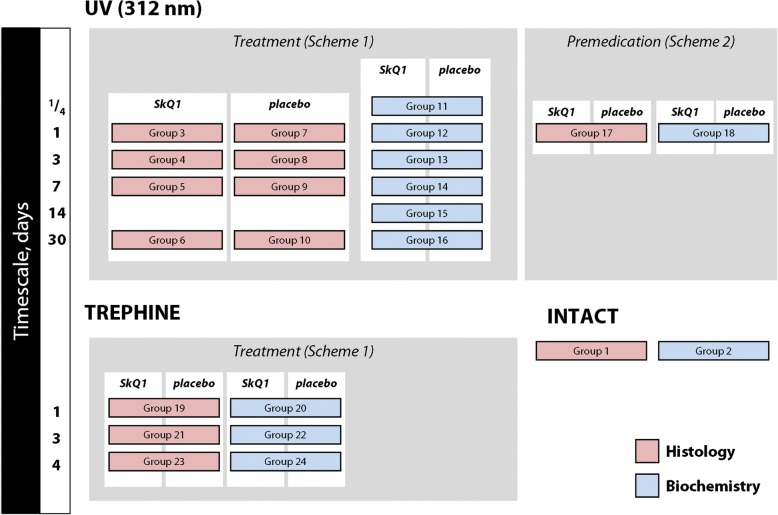
Graphical representation of the division of groups. The detailed setup of models and treatment regimen are described in the Methods section

The division of groups is presented schematically in Fig. 1. Rabbits were separated into 24 groups of 6 animals. Groups 1 and 2 contained intact animals and they were used as reference for clinical/histological and biochemical studies, respectively. Rabbits from groups 3–18 were exposed to UV irradiation and groups 19–24 were subjected to trephine scarification. The animals from groups 3–10 were treated (Scheme 1) with SkQ1 (groups 3–6) or placebo (groups 7–10) in both eyes. Rabbits were sacrificed on days 1 (groups 3, 7), 3 (groups 4, 8), 7 (groups 5, 9) and 30 (groups 6, 10), and their corneas were subjected to histological analysis. Animals from groups 11–16 were treated (Scheme 1) with SkQ1 (left eye) or placebo (right eye). The corneas were harvested 6 h (group 11) and 1 (group 12), 3 (group 13), 7 (group 14), 14 (group 15) and 30 days (group 16) after the final UV irradiation session and used for biochemical studies. The animals from groups 17 and 18 were premedicated (Scheme 2) with SkQ1 (left eye) or placebo (right eye). Their corneas were extracted on the next day after the final irradiation session and used for histological (group 17) or biochemical (group 18) analysis. The trephined animals (groups 19–24) were treated (Scheme 1) with SkQ1 (left eye) or placebo (right eye), sacrificed on day 1 (groups 19, 20), 3 (groups 21, 22) and 4 (groups 23, 24) after the operation and their corneas were used for histological (groups 19, 21, 23) and biochemical (groups 20, 22, 24) analysis.

### Clinical examination of cornea

The development of corneal injury was monitored by fluorescein staining of the ocular surface as described in [24]. The registered corneal injuries were assigned clinical scores of 0–6 depending on the size of the affected corneal surface: no fluorescein staining (0 points), staining of 0–12.5% of the corneal surface (1 point), 12.5–25% (2 points), 25–50% (3 points), 50–75% (4 points), 75–90% (5 points) and > 90% (6 points). Mean clinical scores were derived by adding the clinical scores for all eyes of the animals in a group and dividing by the number of eyes ± standard error (SE).

### Histological analysis

The fixation and examination of the cornea was performed as described in [24]. Briefly, the eyeballs were enucleated immediately post-mortem and fixed in 10% neutral buffered formalin in PBS (pH 7.4) for 24 h at room temperature or in Carnoy’s solution (60% ethanol, 30% chloroform, and 10% glacial acetic acid) for 3 h at room temperature. The corneas were trimmed out of fixed eyeballs, dehydrated and embedded in paraffin medium*.* Eight three-micron-thick nasotemporal cross-sections from anterior to posterior surfaces through peripheral and central areas of each cornea (at 300-μm intervals) were prepared. Corneal sections were stained with hematoxylin and eosin and examined using Axio Scope.A1 microscope (Carl Zeiss, Germany) and Leica DM400 (Leica, Germany) microscopes. Microphotographs were obtained by an AxioCam MRc 5 megapixel color camera (Carl Zeiss) and processed using AxioVision v.3.0 (Carl Zeiss) and Photoshop CS3 software (Adobe systems, USA).

### Corneal samples

Full-size rabbit corneas were excised, placed into 400 μl of PBS, and frozen at − 70 °C. After thawing, the tissue was sonicated for 10 min on ice. Corneal extracts for biochemical evaluations were obtained by centrifugation of the samples (15,000 g, 10 min) at + 4 °C. The supernatants were aliquoted and stored at − 70 °C, and the pellets were homogenized in MDA lysis buffer (Sigma-Aldrich) and used for MDA measurements as follows.

### Malondialdehyde assay

MDA concentration was measured in corneal homogenates by thiobarbituric acid assay using commercially available kit (Sigma-Aldrich). Intensity of colorimetric reaction at 532 nm was determined using Synergy H4 Hybrid Reader (Biotek, USA). The data were analyzed using SigmaPlot 11 (SYSTAT Software, USA).

### Total protein concentration

Protein concentration in corneal extracts was measured by the bicinchoninic acid (BCA) assay using commercially available kit (Thermo Fisher Scientific, USA) in accordance with the manufacturer’s instructions. Intensity of colorimetric reaction at 562 nm was determined using Synergy H4 Hybrid Reader. The data were analyzed using SigmaPlot 11.

### Total antioxidant activity

The corneal extracts were analyzed using standardized hemoglobin/H_2_O_2_/luminol model system [25]. Standard solutions, containing 1–8 μM Trolox (6-hydroxy-2,5,7,8-tetramethylchroman-2-carboxylic acid) in PBS, were used as reference and total antioxidant activity (AOA) was expressed in Trolox equivalent. Luminol oxidation reaction in the model system was stimulated by the addition of hydrogen peroxide to final concentration of 6 μM, after which chemiluminescence was registered each 1 s for 10 min using Glomax-Multi Detection System luminometer (Promega, USA). The data were analyzed using SigmaPlot 11.

### Activity of antioxidant enzymes

The activity of superoxide dismutase (SOD) and glutathione peroxidase (GPx) was evaluated in the corneal extracts, using commercially available kits (Sigma-Aldrich, Randox) in accordance with the manufacturer’s instructions. Intensity of colorimetric reactions was determined using Synergy H4 Hybrid Reader or Ultrospec 1000 (Pharmacia, Sweden). The acquired data were analyzed using SigmaPlot 11. The activity of the enzymes in corneal extracts was normalized to 1 mg of the total protein.

### Statistics

The data were analyzed by the mean standard error (SE) method. Mean, SE, and statistical significance were calculated using SigmaPlot 11. Statistical significance was assessed using unpaired two-tailed t-test for the experiments where SkQ1 and placebo were administered to separate groups, and paired two-tailed t-test for the experiments where every animal received SkQ1 in their left eye and placebo in their right eye. The probability of 0.05 was considered significant.

## Results

### Clinical characterization of the corneal state after UV irradiation and scarification with or without SkQ1 premedication/treatment

**Fig. 2:**
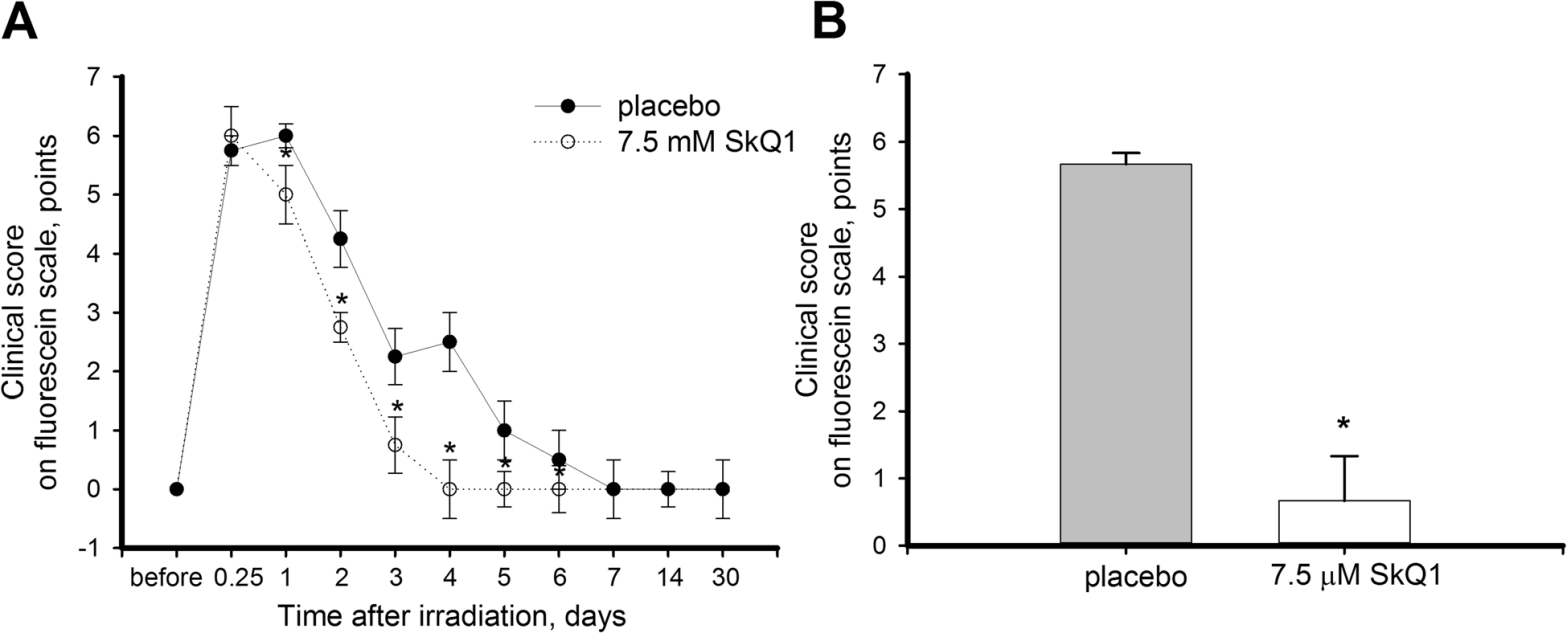
Clinical state of cornea after UV irradiation upon treatment or premedication with SkQ1. Rabbits eyes were exposed to 312 nm UV irradiation (halogen lamp, 8 W) for 4 days, 20 min per day with or without treatment (**a**) or premedication (**b**) with 7.5 μM SkQ1 eye drops. Clinical score on a fluorescein scale (0–6 points) was determined daily for 30 days (**a**) or on the next day (**b**) after the last irradiation session. *p < 0.05 compared with the values measured in control samples

In the UV damage model, the rabbit eyes were exposed to intense irradiation with 312 nm light, 20 min per day for 4 days. Corneal state was evaluated immediately after the exposure and then monitored daily for 30 days by fluorescein staining. The obtained clinical data indicate that UV irradiation induced prominent corneal lesions: in 6 h following the final exposure the score of corneal damage in affected animals reached 5–6 points on fluorescein scale and persisted on this level during the next 24 h (Fig. 2a). The corneal healing started at 48 h and by day 7 no fluorescein staining was observed, indicative of complete re-epithelialization of the cornea.

Post-exposure treatment with conjunctival instillations of 7.5 μM SkQ1 significantly improved corneal healing rates in experimental animals. No corneal lesions (score 0–0.5) were detected by fluorescein staining as soon as on day 4 of postoperative period, as opposed to the animals that received placebo (Fig. 2a). Premedication with 7.5 μM SkQ1, in turn, efficiently protected corneas from UV damage (Fig. 2b). The eyes received the antioxidant premedication displayed either none or mild (0–2 points on fluorescein scale) corneal erosion already on the first day after the irradiation, indicative of high level of epitheliocyte survival in contrast to the control eyes, where severe corneal damage (up to 6 points on fluorescein scale) was observed. Thus, according to clinical examination, SkQ1 possesses potent therapeutic action in respect to UV-damaged cornea and effect of the antioxidant is more prominent when it is used as premedication than in the case of its post-exposure administration.

**Fig. 3:**
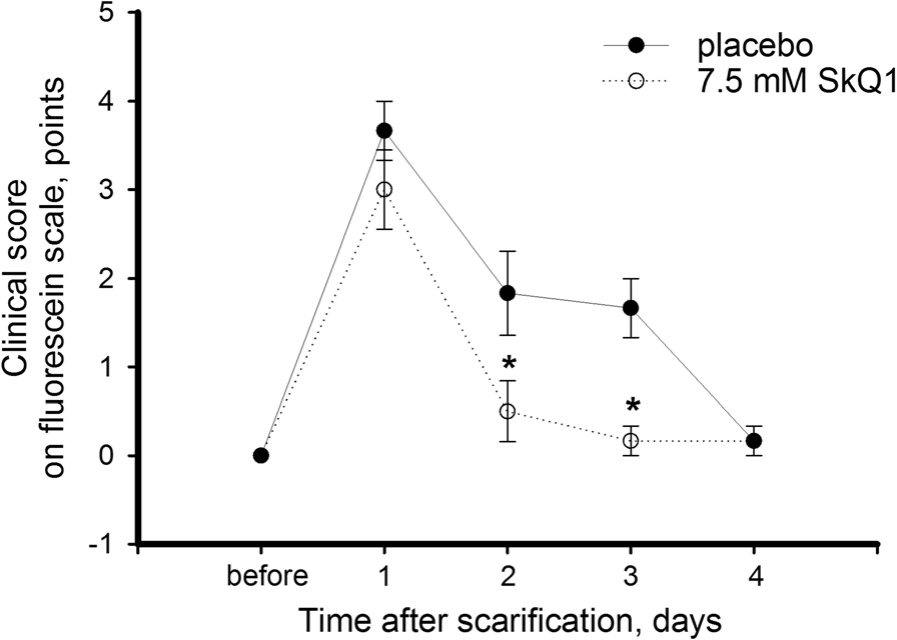
Clinical state of cornea after trephine scarification upon treatment with SkQ1. Rabbit eyes were scarified with trephine knife (7 mm in diameter, 50 μm deep) with or without treatment with 7.5 μM SkQ1 eye drops. Clinical score on a fluorescein scale (0–6 points) was determined daily for 4 days after the operation. *p < 0.05 compared with the values measured in control samples

In the mechanical injury model, the dynamics of corneal regeneration after 50 μm-deep incisions with a trephine knife was monitored on clinical level daily for 4 days. During this time period, the right eyes of the experimental animals were treated with placebo, while the left eyes received instillations of 7.5 μM SkQ1. Fluorescein tests revealed that without treatment full re-epithelialization of the injured cornea occurred on day 4 after the scarification, which is significantly faster than in case of UV-derived injury (Fig. 3). Meanwhile, treatment with SkQ1 accelerated corneal regeneration in experimental animals, allowing achieving almost full recovery after 2 days of therapy. These data demonstrates that mitochondria-targeted antioxidant therapy using SkQ1 enhances corneal wound healing after the mechanical injury.

### Histological examination of the cornea after UV irradiation and scarification with or without SkQ1 premedication/treatment

**Fig. 4:**
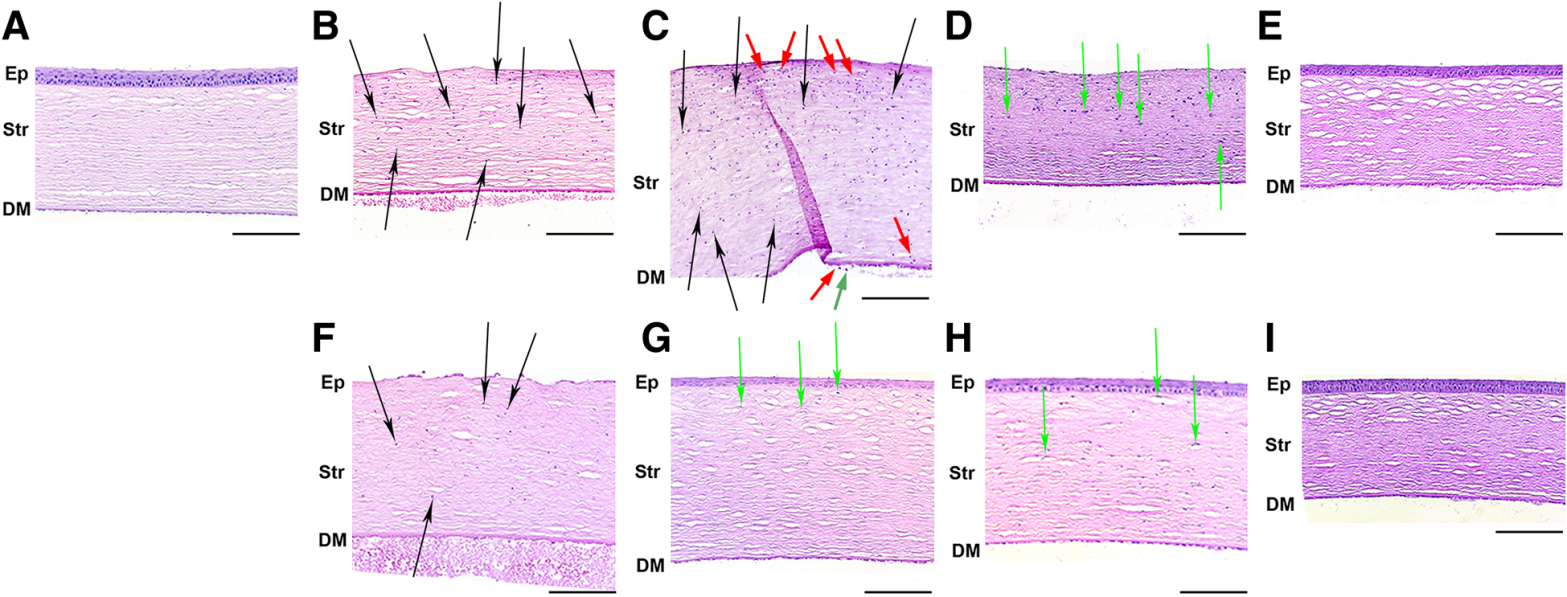
Morphology of cornea after UV irradiation upon treatment with SkQ1. Representative microscopic images of hematoxylin and eosin staining of normal rabbit cornea (**a**) or rabbit corneas after irradiation with 312 nm UV light (halogen lamp, 8 W) for 4 days, 20 min per day (**b**-**i**). Corneas without treatment on the 1st (**b**), 3rd (**c**), 7th (**d**) and 30th (**e**) day after the last irradiation session. Corneas treated with daily instillations of 7.5 μM SkQ1 on the 1st (**f**), 3rd (**g**), 7th (**h**) and 30the (**i**) day after the last irradiation session. Ep - corneal epithelium (if the designation Ep is absent, a complete loss of corneal epithelium is observed in preparation), Str - stroma, DM - Descemet’s membrane. Pyknotic changes (apoptosis) in keratocytes are shown by black arrows. Granulocytic infiltration in stroma and anterior eye chamber is shown by red arrows. Green arrows are for activated collagen-synthesizing keratocytes. Due to abundance of pyknotic nuclei, granulocytes and activated keratocytes in corresponding slides, not every one of them is labeled. Magnification ✕200, scale bar 100 μm

To monitor effects of SkQ1 on corneas on cellular level, the rabbit eyes were subjected to histological analysis. In the case of UV damage model, without treatment at 24 h post exposure all animals displayed signs of persistent corneal injury such as denudation of the basal membrane (complete loss of corneal epithelium) in the central portions of cornea, pyknotic changes (apoptosis) in keratocytes (Fig. 4b, black arrows) as well as pyknosis and vacuolization of endothelial cells. In wound periphery, early signs of regenerative process, such as formation of epithelial rolls on wound edges and pronounced thinning of epithelial layers in the unaffected areas, were also detected. On day 3, the active phase of epithelialization was observed as activated epitheliocytes migrated centripetally over the wound surface. This was accompanied by a pronounced granulocytic infiltration and hypopyon in the anterior chamber (Fig. 4c). Notably, at this stage there was a general decline in the number of cells in corneal endothelium and the corneas exhibited stromal edema and mass apoptosis of stromal keratocytes (Fig. 4c, black arrows), as well as granulocytic infiltration of the stroma (keratitis, Fig. 4c, red arrows). After 7 days, the re-epithelialization process was mostly completed, but the very central portions of the corneas were still denuded (Fig. 4d), which correlated with the fluorescein test results. Endothelium and Descemet’s membrane displayed normal morphology and corneal stroma continued remodeling as indicated by activation of remaining keratocytes (Fig. 4d, green arrows), signs of collagen synthesis and absence of edema on this point (Fig. 4d). Finally, on day 30 newly formed cornea was indistinguishable from the normal tissue (Fig. 4e).

The treatment of the UV-irradiated corneas by SkQ1 instillations produced a pronounced therapeutic effect on cornea in agreement with clinical observations. While on the first day there was no observable difference between corneas of treated and control animals (Fig. 4f), by day 3 the treated corneas displayed reduced stromal edema, complete epithelialization and active remodeling of the stroma by collagen-producing keratocytes (Fig. 4g, green arrows). On day 7 in experimental group the advanced stage of stroma remodeling was observed, whereas in control group short coarse collagen fibers were still present (Fig. 4h). Furthermore, the rate of keratocyte death and the level of inflammatory infiltration were significantly lower in the treated samples. By day 30 both control and treated corneas were completely healed (Fig. 4i and e).

**Fig. 5:**
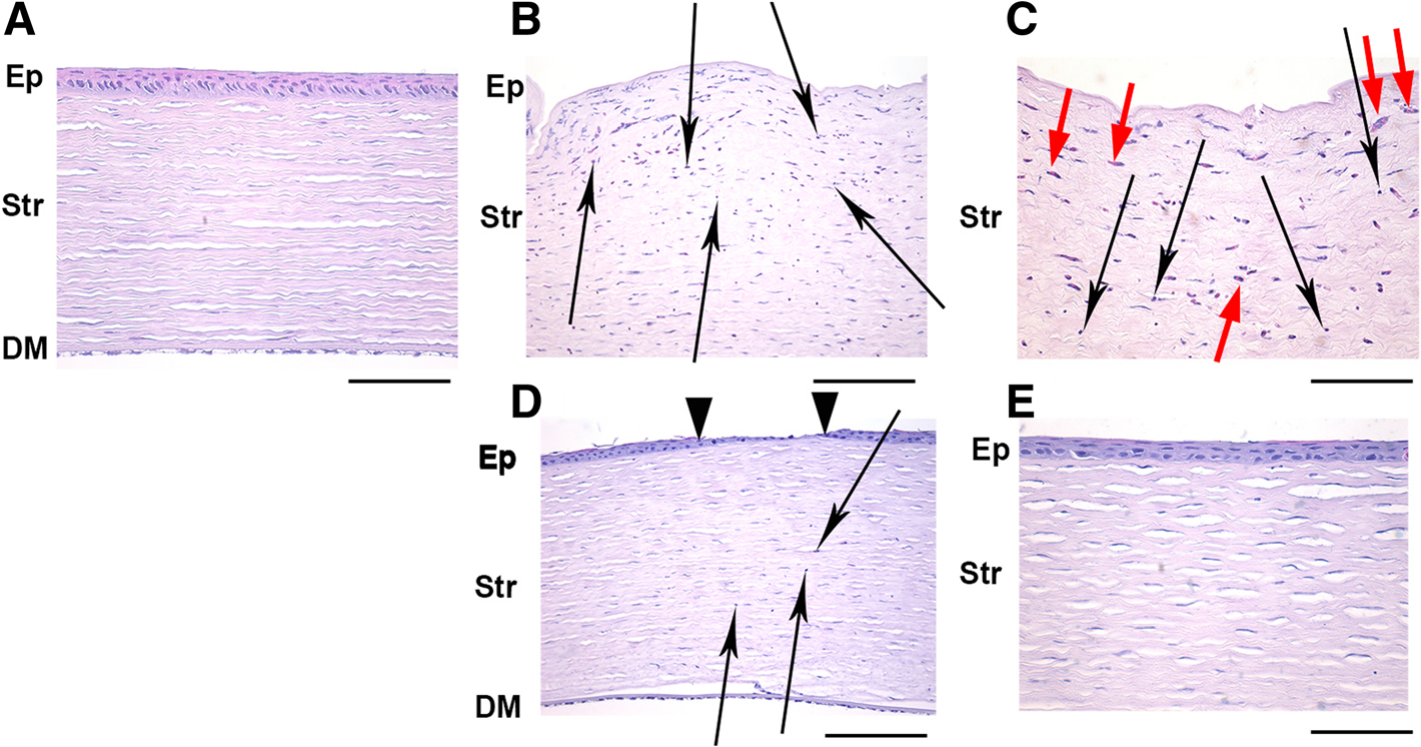
Morphology of cornea after UV irradiation upon premedication with SkQ1. Representative microscopic images of hematoxylin and eosin staining of normal rabbit cornea (**a**) or rabbit corneas after irradiation with 312 nm UV light (halogen lamp, 8 W) for 4 days, 20 min per day (**b**-**e**). Corneas one day after the last irradiation session with (**d**, **e**) or without (**b**, **c**) premedication with 7.5 μM SkQ1. Ep - corneal epithelium (if the designation Ep is absent, a complete loss of corneal epithelium is observed in preparation), Str - stroma, DM - Descemet’s membrane (if the designation DM is absent, the cornea in course of edematous changes became so thick that Descemet’s membrane and endothelium did not not fit in picture size). Pyknotic changes (apoptosis) in keratocytes are shown by black arrows. Granulocytic infiltration in stroma and anterior eye chamber is shown by red arrows. Due to abundance of pyknotic nuclei and granulocytes, not every one of them is labeled). Black arrowheads label an area of decrease in number of epithelial cell layers. Magnification ✕200 (A, B, D), ✕400 (**c**, **e**); scale bar 100 μm (**a**, **b**, **d**), 50 μm (**c**, **e**)

The premedication with SkQ1 was even more beneficial, as it markedly prevented UV-induced epithelium loss. Thus, in placebo eyes on day 1 after the exposure, central portion of corneas exhibited total loss of epithelium due to apoptotic death of epitheliocytes (Fig. 5b). The stroma was edematous and showed signs of inflammation and apoptotic death of keratocytes (pyknotic nuclei) (Fig. 5c, red and black arrows, respectively). By contrast, in SkQ1-premedicated animals, corneal epithelium was much more preserved, and basal membrane denudation was virtually absent (Fig. 5, d-e). Although in some areas there was a slight decrease in number of cell layers due to apoptotic death of epithelial cells (Fig. 5d, black arrowheads), in general, epithelium persisted over a significant surface of the cornea (Fig. 5d). In addition, in premedicated corneas pyknotic (apoptotic) nuclei of keratocytes and activation of keratocytes were less frequent and edema and inflammatory infiltration was significantly less prominent (Fig. 5e). Taken together these data demonstrate that SkQ1 averts or considerably suppresses UV-induced apoptotic death of corneal epithelial cells and keratocytes as well as reduces the level of inflammatory infiltration and edema. The therapeutic effects of the antioxidant are more pronounced in the case of its preventive application, in accord with clinical findings.

**Fig. 6:**
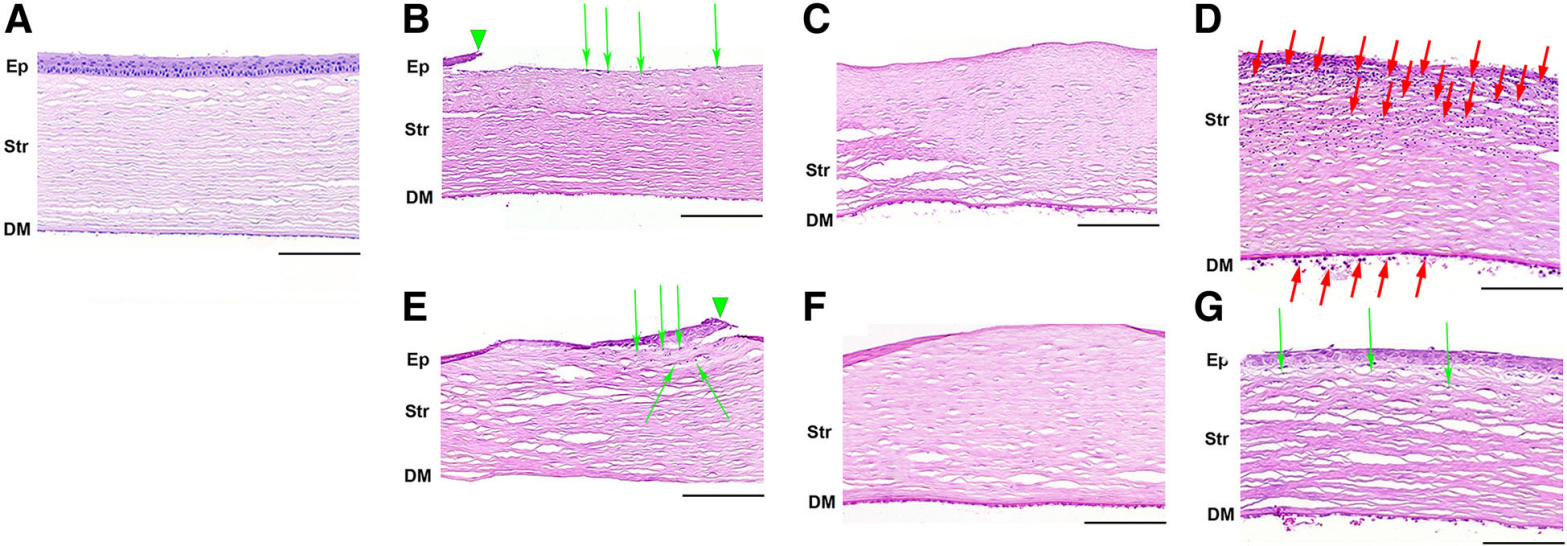
Morphology of cornea after trephine scarification upon treatment with SkQ1. Representative microscopic images of hematoxylin and eosin staining of normal rabbit cornea (**a**) or rabbit corneas scarified with trephine knife (7 mm in diameter, 50 μm deep) (B-G). Corneas without treatment on the 1st (**b**), 3rd (**c**) and 4th (**d**) day after trephination. Corneas treated with daily instillations of 7.5 μM SkQ1 on the 1st (**e**), 3rd (**f**) and 4the (**g**) day after trephination. Ep - corneal epithelium (if the designation Ep is absent, a complete loss of corneal epithelium is observed in preparation), Str - stroma, DM - Descemet’s membrane. Green arrowhead labels margin of migrating activated epithelial roll. Green arrows are for activated collagen-synthesizing keratocytes. Granulocytic infiltration in stroma and anterior eye chamber is shown by red arrows. Due to abundance of granulocytes and activated keratocytes, not every one of them is labeled). Magnification ✕200, scale bar 100 μm

In the case of mechanical injury model, without treatment on day 1 after the scarification the major portion of corneal surface was completely devoid of epithelium (Fig. 6b). Wound edges were marked by epithelial rolls (Fig. 6b, green arrowhead) and activated epithelium with fewer layers. Interestingly, living keratocytes were found throughout the stroma, especially in the region underlying the wound, and in vicinity of epithelial rolls they were activated (Fig. 6b, green arrows). In contrast to UV model, deep stromal layers, Descemet’s membrane and endothelium were intact. On day 3 partial re-epithelialization of the scarified area was observed, although a focus of denuded stroma still presented in the center of cornea, flanked by epithelial rolls (Fig. 6c). Basal membrane between the stroma and the new epithelium was enriched in amorphous extracellular matrix in the foci nearest to the newly formed epithelium. The central area of cornea contained coarse newly-synthesized collagen and there were many activated keratocytes producing this protein. At the same time, pyknotic nuclei (apoptosis) of stromal keratocytes were still prominent and intense inflammatory infiltration was still present both in cornea and anterior chamber (Fig. 6d, red arrows). The complete healing required more than 4 days, which is in agreement with clinical data. By day 4 inflammation ceased as hypopyon was unobservable and anterior chamber contained only a small amount of mildly eosinophilic debris. The postoperative treatment with SkQ1 instillations improved healing rates of the scarified corneas (Fig. 6, e-g). In contrast to placebo-treated samples, by day 4 re-epithelialization was mostly complete, new epithelium was active and mitotic (Fig. 6g); stroma was enriched with active keratocytes (Fig. 6g, green arrows) and interacted with epithelium. In addition, there was significantly less inflammatory exudate found in the anterior chamber. Thus, antioxidant treatment enhanced restoration of corneal epithelium and stroma and reduced corneal inflammation after the scarification thereby accelerating regeneration of the cornea after the mechanical injury.

### Monitoring of oxidative stress in UV and mechanically damaged cornea with or without SkQ1 premedication/treatment

**Fig. 7:**
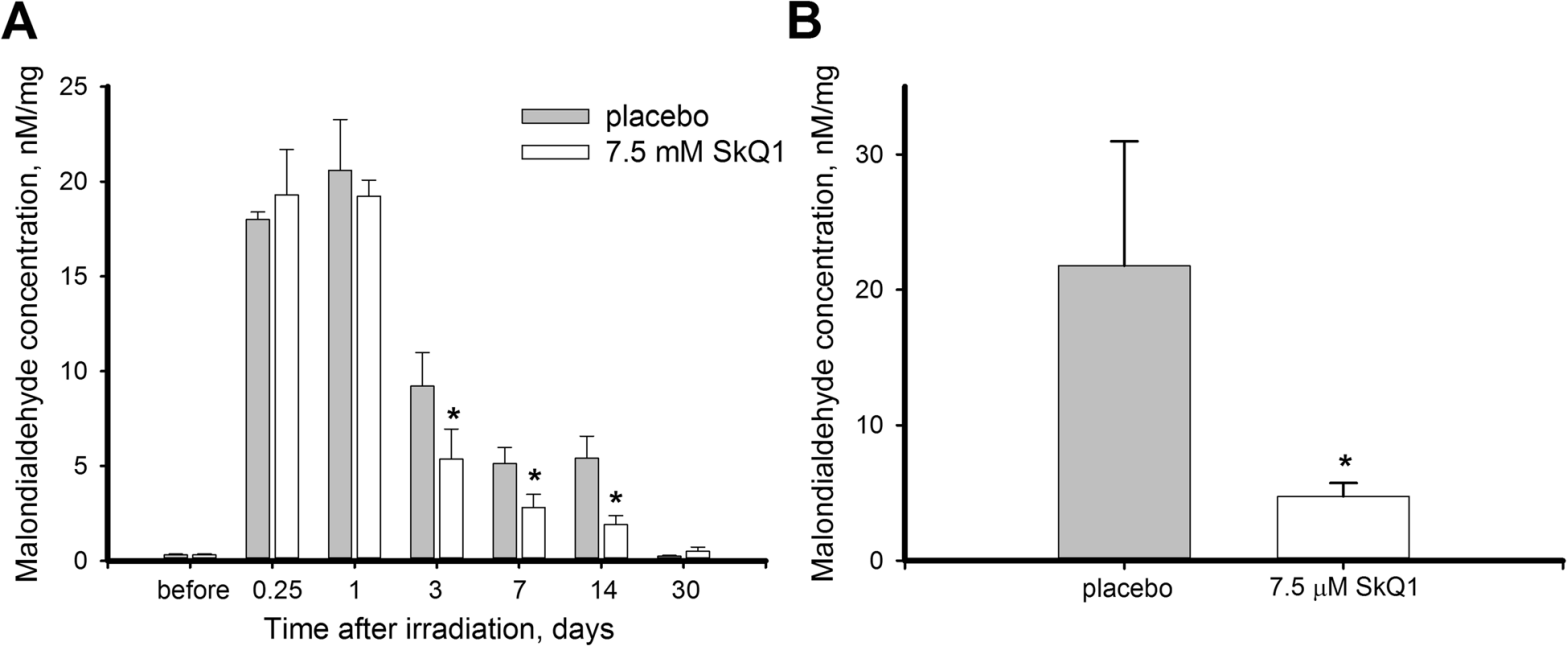
Oxidative stress in cornea after UV irradiation upon treatment and premedication with SkQ1. Concentration of MDA in homogenates of the rabbit corneas exposed to 312 nm UV irradiation (halogen lamp, 8 W) from the distance of 40 cm for 4 days, 20 min per day with or without treatment (**a**) or premedication (**b**) with 7.5 μM SkQ1 eye drops. *p < 0.05 compared with the values measured in control samples

We next monitored intensity of oxidative stress in UV and mechanically damaged rabbit corneas and examined effects of SkQ1 administration under these conditions. To this end, the content of malondialdehyde (MDA) was assayed in the corneal homogenates as this product of lipid peroxidation is commonly regarded as a marker of oxidative stress [17]. MDA determinations were firstly performed with the samples obtained from of UV-exposed animals treated/premedicated with placebo or 7.5 μM SkQ1. According to the data of colorimetric thiobarbituric acid assay (Fig. 7a), MDA concentration in corneas of control eyes exceeded baseline values more than 60-fold after 6 h and remained on this maximum level 24 h after the final irradiation session. This MDA burst was followed by its gradual decline to the normal concentrations during the 30 days of postoperative period.

Antioxidant treatment with 7.5 μM SkQ1 notably accelerated normalization of MDA, although its peak levels remained unaffected. Thus, starting from day 3, the treated corneas displayed approximately 2-fold reduction of MDA content as compared to the control. Interestingly, premedication of corneas with SkQ1 much more prominently mitigated elevation of MDA content after the exposure to UV light (Fig. 7b). Thus, MDA only increased 15-fold in SkQ1 pre-treated rabbits in contrast to 70-fold MDA elevation in animals premedicated with placebo. In the aggregate, these data indicate that the revealed therapeutic effects of SkQ1 on the damaged corneas stems from its high efficacy in suppressing UV-induced oxidative stress of the corneal cells.

**Fig. 8:**
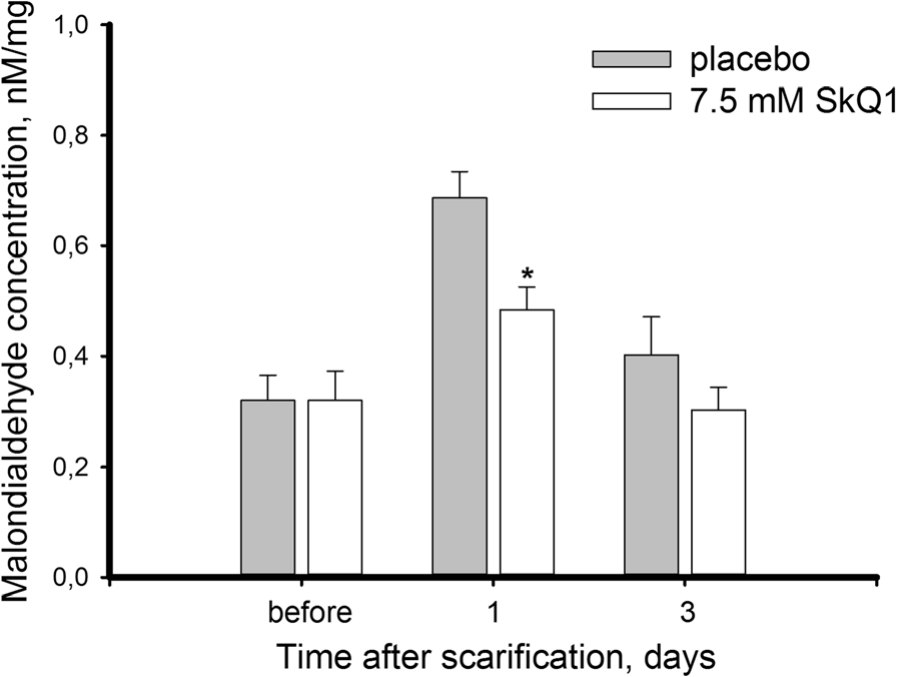
Oxidative stress in cornea after trephine scarification upon treatment with SkQ1. Concentration of MDA in homogenates of the rabbit corneas scarified with trephine knife (7 mm in diameter, 50 μm deep) with or without treatment with 7.5 μM SkQ1 eye drops. *p < 0.05 compared with the values measured in control samples

Unlike the UV damage model, in the scarification model the oxidative stress was moderate as median MDA levels increased only approximately 2-fold (Fig. 8). Nevertheless, the treatment with SkQ1 reduced maximum MDA elevation and accelerated its normalization in trephined cornea. Thus, on day 1 after the operation MDA concentration increased in treated eyes only by 60% and it returned to the normal values within next two days. Despite this noticeable antioxidant action of SkQ1, it seems unlikely that it solely underlies the pronounced acceleration of corneal wound healing, suggesting that some other biochemical effects of the drug facilitated regeneration process.

### Alterations in antioxidant defense system of the cornea under conditions of UV and mechanically induced damage with or without SkQ1 premedication/treatment

**Fig. 9:**
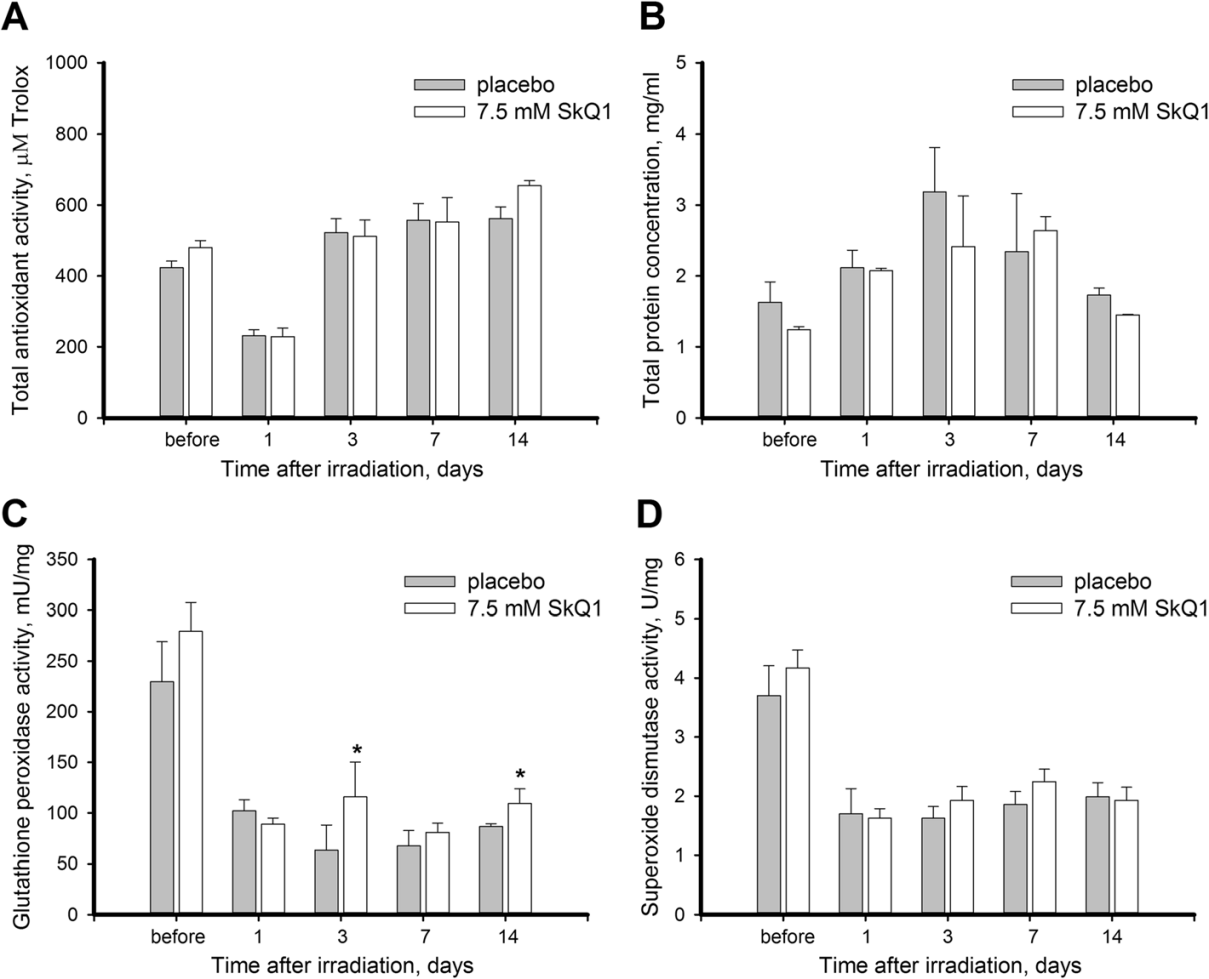
Antioxidant activity in cornea after UV irradiation upon treatment with SkQ1. Total antioxidant activity (**a**), protein concentration (**b**), glutathione peroxidase activity (**c**), and superoxide dismutase activity (**d**) in extracts of thr rabbit corneas exposed to 312 nm UV irradiation (halogen lamp, 8 W) for 4 days, 20 min per day with or without treatment with 7.5 μM SkQ1 eye drops. *p < 0.05 compared with the values measured in control samples

On the final step, we examined functionality of innate antioxidant defense components in rabbit corneas subjected to UV irradiation/scarification and analyzed effect of SkQ1 on these components. In particular, we assessed total AOA provided by low molecular weight antioxidants and analyzed activity of antioxidant enzymes GPx and SOD in the cornea as these components of antioxidant defense were previously shown to be affected in DES [26–28]. For the analysis, corneal extracts were first obtained from UV-exposed animals, treated/premedicated with placebo or 7.5 μM SkQ1. Without treatment, the activity of all these components declined significantly (approximately 2-fold) on day 1 after the final UV irradiation session in agreement with pronounced oxidative stress developed in the cornea at this time point (Fig. 9). Furthermore, GPx continued declining afterwards, retaining only 25% of its normal activity by day 3 (Fig. 9c). Meanwhile, they displayed different recovery profiles: AOA restored after 3 days post exposure (Fig. 9a), whereas the enzymes never fully recovered in the course of the 14-day observation period (Fig. 9, c-d). Notably, antioxidant enzyme activity declined disproportionally to the gradual elevation of total protein content in the corneal extract, which peaked on day 3 and returned to the normal levels by day 14 (Fig. 9b).

**Fig. 10:**
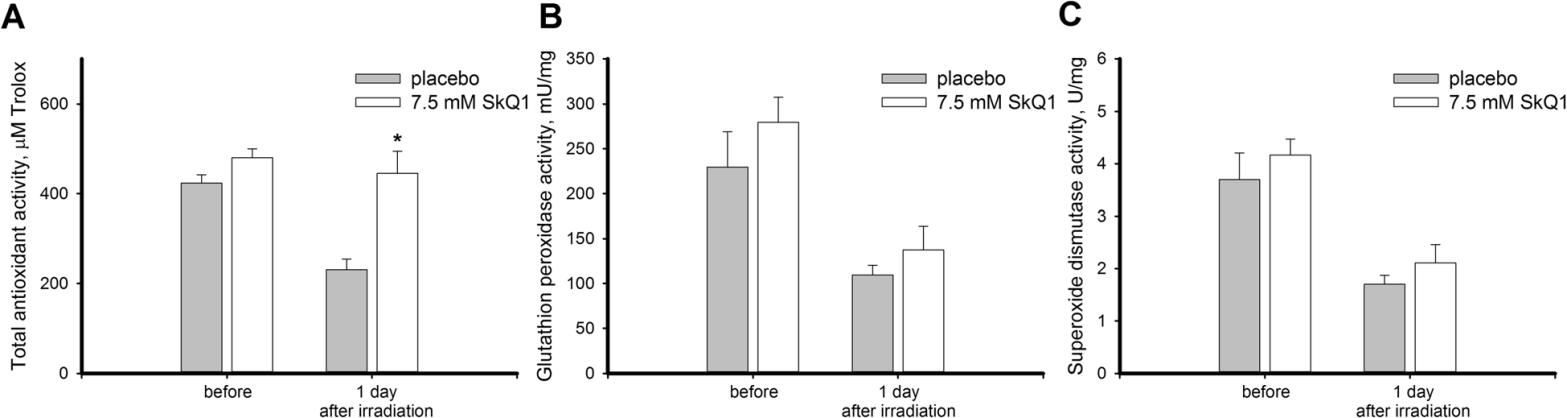
Antioxidant activity in cornea after UV irradiation upon premedication with SkQ1. Total antioxidant activity (**a**), glutathione peroxidase activity (**b**), and superoxide dismutase activity (**c**) in extracts of the rabbit corneas exposed to 312 nm UV irradiation (halogen lamp, 8 W) for 4 days, 20 min per day with or without premedication with 7.5 μM SkQ1 eye drops. *p < 0.05 compared with the values measured in control samples

The treatment with SkQ1 did not affect either AOA or SOD activity in the damaged corneas and provided minor, but reproducible effect on GPx: in treated animals GPx activity was 80% higher than in control animals on day 3, and 25% higher than in control animals by the end of the observation period (Fig. 9c). By contrast, premedication with SkQ1 produced a prominent effect on AOA completely preventing its UV-induced reduction in the corneas (Fig. 10a). In addition, premedicated corneas were characterized by slightly higher GPx and SOD activities as compared to control, although this effect was not found to be statistically significant (Fig. 10, b-c). The revealed changes in antioxidant defense (especially in the case of AOA) correlated with the intensity of the ongoing oxidative stress in the damaged corneas (see Fig. 7). Consistently, the pronounced effect of SkQ1 in prevention of oxidative stress (see Fig. 7b) resulted in maintaining normal AOA levels in the premedicated cornea (Fig. 10a).

**Fig. 11:**
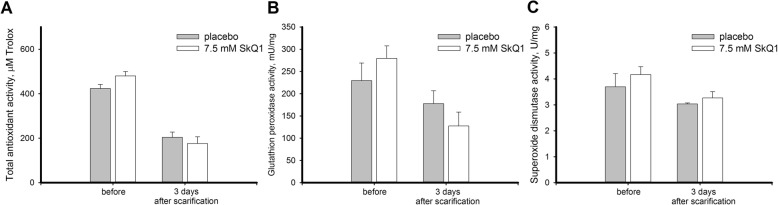
Antioxidant activity in cornea after trephine scarification upon treatment with SkQ1. Total antioxidant activity (**a**), glutathione peroxidase activity (**b**), and superoxide dismutase activity (**c**) in extracts of the rabbit corneas scarified with trephine knife (7 mm in diameter, 50 μm-deep) with or without treatment with 7.5 μM SkQ1 eye drops

In the mechanical injury model, AOA of the rabbit corneas decreased approximately 2-fold in 24 h after scarification (Fig. 11a), which is in agreement with evolved albeit moderate oxidative stress at this time point. However, there was no reproducible effect of scarification on GPx and SOD activity in the corneas (Fig. 11, b-c). Consistently, neither of these antioxidant components was reliably affected by SkQ1 treatment.

Collectively, our biochemical data demonstrate that the revealed protective effect of SkQ1 on the cornea in the UV damage model is associated with the suppression of intense oxidative stress and restoration of AOA of this tissue. In the case of mechanical injury model, the oxidative stress in the cornea, although inhibited by SkQ1, is initially less pronounced, and therefore the effect of the antioxidant is likely supported by its additional biochemical activities.

## Discussion

Cornea is the barrier protecting the eye from natural and anthropogenic UV irradiation. Meanwhile, in popular forms of photorefractive surgery, namely LASIK and PRK, cornea is exposed to relatively high doses of UV irradiation coming from excimer lasers, which were reported to induce persistent corneal lesions and mild keratitis [29–32]. Furthermore, in PRK, the exposure of the cornea to UV irradiation is preceded by mechanical removal of significant part of corneal epithelium (in contrast to LASIK, where epithelium flap is peeled off using precise cuts made by infrared laser and is put in place afterwards), which increases the risk of complications due to longer recovery times. In addition, refractive surgery weakens the protective qualities of the corneal epithelium and makes it more vulnerable to UV irradiation from natural sources. Consistently, the patients are recommended to wear sunglasses and apply anti-inflammatory and antioxidant agents for several months after the operation to avoid complications [33]. In this study we attempted to model all potentially harmful components of excimer laser photorefractive surgery and trial novel mitochondria-targeted antioxidant therapy for prevention of postoperative adverse effects and improvement of corneal wound healing. To this end, we used experimental models of UV and mechanical corneal injury in rabbits. These species are well suited for ophthalmological research due to many similarities in biomechanical properties of rabbit and human eyes [34]. Indeed, in our previous works rabbits were successfully employed for monitoring anesthesia-induced damage and oxidative stress in cornea on clinical, histological and biochemical levels [24, 27, 28].

In our studies, we employed irradiations of the cornea with 312 nm UV light. In general, cornea is highly susceptible to damage induced by a wide range of UV wavelengths. Therefore, the established animal models of UV-induced corneal damage include all three ranges of UV irradiation, namely UVA (315–400), UVB (280–315 nm) and UVC (100–280). Meanwhile, UVB spectrum was shown to have the highest damaging potential: in doses as small as 0.5 J/cm^2^ it can lead to significant alterations in corneal epithelium and affect the optical properties of the tissue [35–37]. Furthermore, long wavelength UVB (312 nm) threatens the non-renewable cells of the corneal endothelium and stroma [38]. In early studies, excimer lasers of the UVB range were widely trialed for photorefractive surgery. Yet, these lasers were found to produce harmful side effects on the cornea, and they were outcompeted by lasers of the UVC spectrum (193 nm) [8–10]. However, the molecular mechanisms of corneal damage induced by UVB and UVC are similar [39–41]. Since the main objective of our study was to evaluate the effectiveness of antioxidant therapy and premedication in the worst-case scenario, more severe UVB model was considered well suited for these purposes. Another benefit of such model is that it additionally simulates post-operative complications induced by the exposure of the ablated cornea to sunlight. The cumulative dose of UV irradiation received by the animals was 0.36 J/cm^2^ per day (1.44 J/cm^2^ total over 4 days). This exposure induced clinically prominent corneal lesions. The damage manifested as complete destruction of corneal epithelium, including its basal layer, which contains dividing cells, crucial for corneal regeneration [42]. Other morphological features included mass apoptosis of keratocytes and corneal endothelium cells, stromal edema and inflammatory cell infiltration. The complete recovery on the clinical level was achieved after 7 days while morphological signs of pathology were only resolved after 30 days. Overall, our model reproduced the key features of long-healing UV-induced injuries described in previous studies [43], which generally corresponded to epithelial defects observed on corneal surface of some PRK and LASIK patients during the postoperative period [44–48].

In turn, mechanically induced corneal lesions were simulated by performing experimental corneal scarification. Namely, 50 μm-thick flap of corneal epithelium was removed with circular trephine knife, imitating the procedure involved in PRK. In this model, despite the complete absence of the epithelium in the damaged area, basal membrane and deeper layers of cornea were mostly unaffected; there were many survived and active keratocytes and the endothelium was intact. In general, our model reproduced the mechanical damage to cornea, associated with epithelium displacement, which was shown to contribute to development of persistent corneal defects in patients undergoing similar surgeries [47, 48].

Both models were used for trialing mitochondria-targeted antioxidant therapy for preventing complications and improving corneal healing in postoperative period. Indeed, oxidative stress was recognized as the most severe damaging factor in UV-induced and other types of corneal injury. ROS are known to mediate lipid peroxidation in corneal epithelium exposed to UV light, with generation of MDA [49]. Consistently, in our UV model, concentration of MDA in irradiated corneas increased more than 60-fold. Remarkably, it did not fully normalize until after 30 days following the final UV irradiation session, long after the clinical signs of superficial epithelial damage were resolved. Thus, the oxidative damage might be responsible for delayed recovery of internal epithelium layers and other corneal cells as well as late resolution of corneal edema and inflammation. Oxidative stress was shown to cause keratocyte apoptosis and reduce the natural re-epithelialization rate, which potentially results in irreversible damage to cornea [50–52]. Indeed, in contrast to the UV model, in corneal scarification model MDA increased only 2.5-fold, which could explain keratocyte survival and preservation of corneal endothelium, as well as faster tissue regeneration.

UV-induced oxidative stress leading to apoptosis of corneal epitheliocytes and other corneal cells is mediated by mitochondrial damage [12, 53, 54]. Indeed, mitochondria are highly sensitive to oxidative stress as they represent intracellular source of ROS and their function depends on redox state of the cell [11, 12]. With this in mind, we applied mitochondria-targeted antioxidant SkQ1 in attempt to prevent or treat the UV-induced oxidative damage. The postoperative therapy with conjunctival instillations of SkQ1 was found to suppress cell death in all corneal layers and accelerate regeneration of the epithelium almost 2-fold. Furthermore, the progress of regeneration strongly correlated with faster resolution of oxidative stress in the treated corneas as indicated by MDA levels. Interestingly, SkQ1 was even more beneficial when administered as premedication. In this case, the development of epithelial lesions on day 1 after the final irradiation session was almost completely prevented both in the center and at the periphery of the cornea, while in the eyes subjected to post-exposure treatment focal epithelial loss still occurred. Furthermore, the histological signs of edema and inflammatory infiltration were also less pronounced in the cornea of the premedicated animals, as compared to the treatment administration scheme. Consistently, the oxidative stress in premedicated animals was weaker as evidenced by lower MDA levels after the UV irradiation sessions. In the case of scarification model, we did not employ SkQ1 premedication because the model involves mechanical removal of a significant portion of antioxidant-loaded tissue right after its administration. Meanwhile, the postoperative therapy with SkQ1, assessed in this case, combines the benefits of both premedication and treatment since it is applied immediately after scarification as distinct from the UV model where SkQ1 is administrated only after 4 days after the initial intervention. Even against moderate oxidative stress observed upon corneal scarification, the effect of SkQ1 was still noticeable, as the treatment resulted in 30% lower MDA level on the 1st day. Overall, SkQ1 is more effective, when used as preventive care, which is consistent with the conclusions of our previous work related to anesthesia-induced corneal abrasions [24]. Taken together, our data clearly demonstrates that SkQ1 can effectively avert and/or heal oxidative damage of the cornea, the main factor inducing postoperative complications after photorefractive surgery.

It is known that SkQ1 specifically targets mitochondria and prevents these organelles from intoxicating the cell with ROS [55]. Thus, the efficacy of SkQ1 action directly depends on amount of the living cells in cornea at the beginning of the antioxidant administration, which explains prominent benefits of the premedication scheme. What are in this case the mechanisms underlying efficacy of post-exposure treatment with SkQ1 in enhancing of corneal wound healing after UV damage? By the time the treatment starts the corneal epitheliocytes are absent due to UV-induced apoptosis. Therefore, antioxidant treatment protects only the cells that survived the initial UV exposure, such as keratocytes and endothelial cells. It is well recognized that UV damage of the cornea includes massive loss of keratocytes that are key participants of corneal regenerative mechanisms and producers of collagen [51]. The hazard of the UVB spectrum also relies on its ability to affect corneal endothelium [17, 41, 56, 57], which is responsible for nutrition of the cornea and maintaining its optical properties [58–60]. Notably, adult endotheliocytes are unable of division, so that dead and injured cells cannot be replaced [57]. Both keratocytes and endotheliocytes possess high metabolic rates and are thereby highly susceptible to mitochondrial damage associated with excessive ROS production [61]. Thus, the suppression of mitochondrial oxidative stress in survived keratocytes and endothelium by SkQ1 treatment might improve their longevity and functional activity during the energy-consuming process of wound healing.

In attempt to understand biochemical mechanisms underlying survival of corneal cells promoted by SkQ1, we analyzed the dynamics of innate antioxidant activity in the UV and mechanically damaged tissue. Firstly, we addressed total antioxidant activity (AOA), provided by low molecular weight antioxidants of the cornea, mainly glutathione, ascorbate, and α-tocopherol. AOA was prominently reduced by UV irradiation, but was restored early in postoperative period. Interestingly, this profile of the AOA alterations, in general, correlated with the amount of the survived epithelial cells. Indeed, corneal epithelium is the main source of low molecular weight antioxidants in cornea [62, 63]. Consistently, AOA was not affected by the SkQ1 treatment, but it was retained in SkQ1-premedicated corneas, which preserved their epithelium. Thus, we propose that antioxidant action of SkQ1 is reinforced by maintaining the innate antioxidant defense in the cornea provided by the epitheliocyte survival.

Secondly, we monitored activity of two major antioxidant enzymes, superoxide dismutase (SOD) and glutathione peroxidase (GPx). SOD is responsible for superoxide anion disproportionation into hydrogen peroxide and molecular oxygen, whereas GPx acts as a scavenger of intracellular hydrogen peroxide, protecting lipids from oxidation [64–66]. Both enzymes were reported to undergo UV-induced alterations [51, 67, 68]. Consistently, they were downregulated in our UV model. Interestingly, this downregulation occurred simultaneously with overall elevation of total protein content in corneal samples, despite the complete loss of epithelium, which normally amounts for 80% of the soluble corneal protein [69]. This discrepancy can be explained by pronounced inflammation, observed in our histological studies, and subsequent contamination of the samples with excess of serum proteins. GPx was more susceptible to UV irradiation than SOD. This agrees with previous observations [51, 67, 68] and is commonly speculated to be caused by loss of GPx-producing cells in corneal endothelium [64]. Consistently, the lowest value of GPx activity was observed on day 3 of the post-exposure period, when the most pronounced apoptotic changes in corneal endothelium occurred. In general, neither of the examined enzymes exhibited prominent susceptibility to SkQ1 premedication or therapy. GPx activity was slightly enhanced by SkQ1 treatment, which could be explained by better preservation of endothelial cells. Similar enhancement was observed in another model of corneal damage produced by general anesthesia [24], although in this case the effect was much more pronounced. Regardless of treatment, GPx and SOD did not recover even when the UV-damage of the epithelium was completely healed, which contrasts with the above described AOA dynamics. We speculate that UV-induced decrease of the antioxidant enzymes activity could be in part a result of the specific regulation of protein expression by UV-dependent signaling pathways known as UV-response [70–72]. Although SkQ1 does not seem to directly affect these mechanisms, it effectively compensates for the decrease in the antioxidant enzyme activity by suppressing mitochondrial oxidative stress and promoting cell survival in the irradiated cornea.

## Conclusions

In this study mitochondria-targeted antioxidant therapy using 7.5 μM SkQ1 was recognized as a promising approach to treating chronic corneal damage, associated with UV irradiation and mechanical injury. Prophylactic administration of SkQ1 efficiently prevented UV-induced damage of all corneal cells including epitheliocytes, whereas the post-exposure treatment with this antioxidant promoted faster restoration of normal corneal metabolism and enhanced corneal wound healing through inhibition of keratocyte apoptosis and endothelial damage. In both administration schemes, SkQ1 possessed cell protector activity via suppressing mitochondrial oxidative stress induced by UV light and mechanical injury. Meanwhile, the most prominent benefit of SkQ1 towards UV-induced damage was observed upon its preemptive application, as in this case effect of the antioxidant is facilitated by innate antioxidant defense of the cornea preserved due to massive survival of epithelial cells. Conveniently, ophthalmic surgeries involving risk of UV-induced and mechanical corneal injury are usually scheduled in advance. Therefore, we propose the SkQ1 premedication in patients undergoing these surgeries to improve corneal regeneration and prevent persistent corneal defects.

## Abbreviations

AOA: total antioxidant activity
GPx: Glutathione peroxidase.
LASIK: Laser-assisted in situ keratomileusis
PBS: Phosphate buffer saline
PRK: Photorefractive keratectomy
SkQ1: Plastoquinonyl-decyl-triphenylphosphonium bromide
SOD: Superoxide dismutase
UV: Ultraviolet
